# Negative impacts of ovarian endometrioma on preantral follicle development: implications for endometriosis-related infertility

**DOI:** 10.3389/fendo.2026.1679042

**Published:** 2026-05-11

**Authors:** Makoto Orisaka, Aya Shirafuji, Natsumi Shimizu-Mizuno, Miki Uesaka, Yuko Fujita, Katsutoshi Mizuno, Masayuki Fujita, Yumiko Miyazaki, Toshimichi Onuma, Hideaki Tsuyoshi, Tetsuya Mizutani, Benjamin K. Tsang, Yoshio Yoshida

**Affiliations:** 1 Department of Obstetrics and Gynecology, Faculty of Medical Sciences, University of Fukui, Fukui, Japan; 2 Department of Cell Biology and Biochemistry, Division of Medicine, Faculty of Medical Sciences, University of Fukui, Fukui, Japan; 3 Department of Nursing, Faculty of Nursing and Welfare Sciences, Fukui Prefectural University, Fukui, Japan; 4 Inflammation and Chronic Disease Program, Ottawa Hospital Research Institute, Ottawa, ON, Canada; 5 Departments of Obstetrics and Gynecology & Cellular and Molecular Medicine, University of Ottawa, Ottawa, ON, Canada

**Keywords:** fibrotic remodeling, granulosa and theca cells, infertility, ovarian endometrioma, oxidative stress, preantral follicle development

## Abstract

**Background:**

Endometriosis is a chronic, estrogen-dependent inflammatory disorder and a leading cause of female infertility. Ovarian endometriomas, a common manifestation of endometriosis, are commonly associated with diminished ovarian function; however, the mechanisms underlying endometrioma-induced follicular dysregulation remain poorly understood. Thus, we aimed to determine whether endometrioma fluid (EmF) compromises preantral follicle development via oxidative stress and tissue fibrosis.

**Methods:**

EmF was collected from six patients during laparoscopic cystectomy or transvaginal ethanol sclerotherapy for endometriomas. Large preantral follicles (diameter: 130–160 µm) were isolated from 14-day-old rats and cultured in the presence or absence of 0.5% EmF and 10 ng/mL follicle-stimulating hormone (FSH). Follicular growth, steroidogenesis, and the expression of granulosa cell (GC) and theca cell (TC) markers were evaluated using morphometric analysis, hormone assays, and quantitative real-time PCR. Oxidative stress and fibrosis were assessed by measuring intracellular reactive oxygen species (ROS) levels and by performing immunostaining for fibrosis markers. In addition, the potential protective effects of pharmacological agents, including antioxidants, antifibrotic drugs, iron chelators, and androgens, were investigated.

**Results:**

EmF inhibited GC proliferation, suppressed FSH-induced estradiol production, and downregulated the expression of FSH receptor, anti-Müllerian hormone, and aromatase. Conversely, EmF promoted TC proliferation while downregulating LH receptor expression and androgenic enzyme levels. EmF also increased ROS generation in GC and induced the expression of the fibrotic markers, including transforming growth factor beta 1 and collagen type III, in TC. Androgen supplementation partially restored GC proliferation and FSH receptor expression, whereas antioxidants, antifibrotic agents, and iron chelators showed no significant effects.

**Conclusions:**

EmF disrupts preantral follicle development by inducing oxidative stress in GC and promoting fibrosis in TC, thereby impairing GC–TC crosstalk. These findings reveal a novel pathogenic mechanism underlying endometrioma-associated infertility and underscore the need for therapeutic strategies targeting both oxidative stress and fibrotic remodeling within the ovaries.

## 1 Introduction

Endometriosis is a chronic, estrogen-dependent inflammatory disorder that affects approximately 10–15% of females of reproductive age worldwide and represents one of the principal causes of female infertility. It is estimated that 25–50% of infertile females are diagnosed with endometriosis, whereas 30–50% of women with endometriosis experience infertility (1, 2). The disease is defined by the ectopic implantation and proliferation of endometrial tissue, with the ovary being the most frequent site of lesion development, leading to the formation of ovarian endometriomas (3, 4). Notably, ovarian endometriomas are observed in 17–44% of patients with endometriosis (5). Although the direct impact of endometriomas on folliculogenesis remains debated, histological studies have shown that follicular growth is markedly suppressed in the ovarian cortex adjacent to endometriomas (6). Furthermore, females with endometriomas undergoing ovarian stimulation for assisted reproductive technology (ART) yield fewer oocytes than those without endometriomas (2, 7). These observations suggest that endometriomas directly impair ovarian follicular development; however, the underlying mechanisms remain unclear.

The pathophysiology of endometriosis-associated infertility is multifactorial, involving pelvic adhesion and scarring, altered immune responses and hormonal signaling, ovarian dysfunction, and persistent pelvic inflammation (8, 9). Chronic inflammation promotes the production of pro-inflammatory cytokines and reactive oxygen species (ROS), leading to oxidative stress, fibrotic remodeling, and tissue dysfunction (10). In the ovaries, endometriomas may exacerbate this inflammatory milieu and disrupt normal hormonal signaling (11). Notably, unlike other ovarian cysts, endometriomas lack a cyst capsule (12), allowing cytotoxic components—such as pro-inflammatory cytokines, ROS, and free iron—to diffuse into the surrounding ovarian cortex and adjacent follicles (9). However, the extent to which endometrioma fluid (EmF) directly affects follicular viability and growth remains unclear.

The ovarian follicle, the fundamental unit of female reproduction, comprises an oocyte encased by granulosa cells (GC) and theca cells (TC). Folliculogenesis is a highly orchestrated process that involves the activation of primordial follicles, their progression through primary, secondary, preantral, and antral stages, and ultimately the selection and maturation of dominant follicles for ovulation. This process is regulated by pituitary gonadotropins—follicle-stimulating hormone (FSH) and luteinizing hormone (LH)—in concert with intraovarian factors, including steroids, growth factors, and cytokines (13). More than 99% of follicles undergo atresia without reaching ovulation. The preantral-to-early antral transition constitutes a pivotal checkpoint that determines follicular survival versus atresia. Preantral follicles that fail to acquire FSH responsiveness at this stage are especially vulnerable to atretic signals (14, 15); consequently, disruptions at this stage can profoundly affect ovarian folliculogenesis and fertility potential (16).

In this study, we hypothesized that the presence of ovarian endometriomas disrupts preantral follicle development by impairing GC and TC function during the preantral-to-early antral transition. Using a rat follicle culture model, we evaluated the direct effects of EmF on follicular growth and cellular integrity. Our findings will provide a basis for understanding the mechanisms underlying preantral follicle development in females with ovarian endometriomas.

## 2 Materials and methods

### 2.1 Reagents and materials

Leibovitz’s L-15 medium, α-MEM, bovine serum albumin (BSA), HEPES, insulin–transferrin–selenium–sodium pyruvate supplement (ITS-A), L-glutamine, and ascorbic acid were purchased from Thermo Fisher Scientific (Tokyo, Japan). Polyvinylpyrrolidone (PVP) was obtained from Merck (Darmstadt, Germany). Recombinant human FSH was provided by the National Hormone and Peptide Program (Harbor-UCLA Medical Center, Torrance, CA). Enzyme immunoassay (EIA) kits for estradiol and testosterone were obtained from Cayman Chemical (Ann Arbor, MI). The Power SYBR Green Cells-to-CT Kit and Power SYBR Green PCR Master Mix were purchased from Ambion (Waltham, MA) and Applied Biosystems (Foster City, CA), respectively. PCR primers were synthesized by Eurofins Genomics (Tokyo, Japan). The tissue optical clearing reagent SCALEVIEW-S4 was obtained from FUJIFILM Wako Chemicals (Osaka, Japan). CellROX Green Reagent was purchased from Invitrogen (Waltham, MA). A polyclonal anti-collagen type III (N-terminal, #22734-1-AP) antibody was obtained from Proteintech (Rosemont, IL). N-acetyl-L-cysteine (NAC; antioxidant) and pirfenidone (PFD; antifibrotic agent) were purchased from FUJIFILM Wako Chemicals (Osaka, Japan) and MedChemExpress (Monmouth Junction, NJ). Deferoxamine mesylate (DFO; iron chelator) and dihydrotestosterone (DHT; non-aromatizable androgen) were obtained from Tokyo Chemical Industry (Tokyo, Japan) and Sigma-Aldrich (St. Louis, MO).

### 2.2 Patients and endometrioma fluid collection

This study was approved by the Institutional Ethics Committee of the University of Fukui (approval number: 20240077), and written informed consent was obtained from all participants. Endometrioma fluid (EmF) was collected from six patients during laparoscopic cystectomy or transvaginal ethanol sclerotherapy for endometriomas. The inclusion criteria were as follows: (i) cysts consistent with endometriosis, (ii) absence of malignant features, (iii) a maximum cyst diameter >5 cm, (iv) symptomatic cysts associated with infertility or pain, and (v) accessibility via the transvaginal route for sclerotherapy. The exclusion criteria included (i) any prior surgical or hormonal treatment for endometriomas and (ii) the use of oral contraceptive pills for indications unrelated to endometriosis. The clinical characteristics of EmF donors are summarized in **Supplementary Table 1**.

Collected EmF was pooled, aliquoted, and stored at −80 °C until use. Immediately before use, samples were thawed at 37 °C and centrifuged at 10,000 × *g* for 30 min. The resulting supernatant was sequentially filtered through Microglassfiber syringe filters Type-SYGF (pore size, 1.0 μm; mdi Membrane Technologies, Camp Hill, PA), diluted 1:200 with culture medium, and subsequently passed through Millex PVDF filters (pore size, 0.22 μm; Merck, Darmstadt, Germany) to remove blood components and debris and to obtain EmF working solutions. Microbial testing confirmed the absence of bacterial and fungal contamination.

### 2.3 Isolation and culture of preantral follicles

Animal experiments were conducted in compliance with the NIH Guide for the Care and Use of Laboratory Animals and approved by the University of Fukui Animal Care Committee (protocol numbers R03059, R04047, R05046, and R06034). Female Sprague–Dawley rats were obtained from Sankyo Labo Service Corporation (Tokyo, Japan) and maintained under standard laboratory conditions.

Large preantral follicles were mechanically isolated from excised ovaries of 14-day-old rats in Leibovitz’s L-15 medium supplemented with 1 mg/mL BSA to maintain tissue viability. Follicles were dissected from the ovarian cortex using a 28.5-gauge insulin needle (Becton Dickinson, Franklin Lakes, NJ, USA) under a stereomicroscope (SZX16, Olympus, Tokyo, Japan), ensuring preservation of the follicular basement membrane and TC layer. Only structurally intact, spherical follicles with clearly distinguishable GC and TC compartments were selected.

Follicle diameters (whole follicle and GC layer) were measured along two perpendicular axes using an inverted microscope (IX71, Olympus). The GC layer was defined as the region enclosed by the follicular basement membrane, enabling consistent discrimination from the surrounding TC layer. Only structurally intact follicles with a mean GC-layer diameter of 130–160 μm and one to two well-defined TC layers were selected for culture.

Each follicle was cultured individually in 96-well plates (Sarstedt, Newton, NC; #83.1837.50) in 100 μL α-MEM supplemented with HEPES (2.6 mg/mL), BSA (1 mg/mL), ITS-A (10 mg/mL), L-glutamine (0.4 mg/mL), ascorbic acid (5 μg/mL), penicillin (100 U/mL), streptomycin (100 μg/mL), and PVP (2% w/v), with or without 10 ng/mL FSH and different concentrations of EmF (0.1%, 0.5%, or 1%). Preliminary tests confirmed that 10 ng/mL FSH represented the minimal concentration required for significant preantral follicle growth in this model.

Due to the preserved optical transparency of isolated preantral follicles, follicle growth was monitored noninvasively by daily bright-field imaging, and diameters were analyzed using cellSens Standard software (Olympus). GC volume was calculated based on the region delineated by the basement membrane, whereas TC volume was determined by subtracting GC volume from total follicle volume. Spent media were collected and stored at −20 °C for subsequent steroid analysis.

### 2.4 Steroid hormone assays

Estradiol and testosterone concentrations in spent media were quantified using EIA kits (Cayman Chemical) according to the manufacturer’s protocols. Intra-assay coefficients of variation (CVs) were 10.9% for estradiol and 9.5% for testosterone, whereas inter-assay CVs were 11.7% and 12.5%, respectively. The assay detection limits were 6 pg/mL for estradiol and 10 pg/mL for testosterone.

### 2.5 Quantitative real-time PCR

On day 3 of culture, total RNA was extracted from individual follicles following sonication and reverse-transcribed using the Power SYBR Green Cells-to-CT Kit. Quantitative real-time PCR (qRT-PCR) was performed using Power SYBR Green PCR Master Mix and the StepOnePlus System (Applied Biosystems). The following genes were analyzed: growth differentiation factor 9 (*Gdf9*), FSH receptor (*Fshr*), LH receptor (*Lhcgr*), anti-Müllerian hormone (*Amh*), steroidogenic acute regulatory protein (*Star*), cholesterol side-chain cleavage cytochrome P450 (*Cyp11a1*), 17α-hydroxylase/C17–20 lyase (*Cyp17a1*), aromatase (*Cyp19a1*), transforming growth factor beta 1 (*Tgfb1*), and 18S rRNA (internal control), with primer sequences listed in **Supplementary Table 2**. PCR cycling conditions were as follows: 95 °C for 10 min, followed by 40 cycles of 95 °C for 15 s and 60 °C for 1 min. Relative gene expression was calculated using the 2^−ΔΔCt^ method, normalized to 18S rRNA.

### 2.6 Detection of intracellular oxidative stress

Intracellular ROS levels were assessed on day 2 of culture by incubating follicles with CellROX Green Reagent for 30 min at 37 °C, followed by washing and fixation with 4% paraformaldehyde in PBS (pH 7.4) for 15 min. Nuclei were counterstained with 1 μg/mL DAPI (Nacalai Tesque, Kyoto, Japan). To enable visualization of internal follicular structures, samples were cleared using the SCALEVIEW-S4 reagent for 60 min and imaged with a confocal laser scanning microscope (FV1200, Olympus, Tokyo, Japan).

For compartment-specific analysis, oxidative stress in the GC layer was quantified by measuring CellROX fluorescence intensity using Fiji (ImageJ, NIH). Regions of interest (ROIs) were defined as the area enclosed by the follicular basement membrane, excluding the oocyte, thereby distinguishing the GC compartment from the surrounding TC layer. Mean fluorescence intensity within each ROI was calculated, and background fluorescence was subtracted to obtain corrected values.

### 2.7 Immunofluorescence

To detect fibrosis markers, follicles cultured for 3 days were fixed in 4% paraformaldehyde for 30 min at room temperature. After blocking with 2% horse serum, 1% BSA, and 0.1% Triton X-100, follicles were incubated overnight at 4 °C with rabbit anti-collagen III antibody (1:200, Proteintech). Alexa Fluor 488–conjugated donkey anti-rabbit IgG (H+L) (1:1000; Invitrogen) was used as the secondary antibody. Nuclei were stained with DAPI. After treatment with SCALEVIEW-S4 for 60 min, follicles were imaged using confocal microscopy (FV1200, Olympus).

### 2.8 Statistical analysis

Data are expressed as mean ± SEM from at least three independent experiments. Statistical analyses were performed using GraphPad Prism 8.4.3 (GraphPad Software, La Jolla, CA). Two-group comparisons were performed using Student’s t-test, whereas one-way ANOVA with Tukey’s *post hoc* test was applied for multiple groups. Homogeneity of variances was confirmed prior to analysis. A P value <0.05 was considered statistically significant.

## 3 Results

### 3.1 Effect of endometrioma fluid on *In vitro* growth of preantral follicles

**Figure 1:**
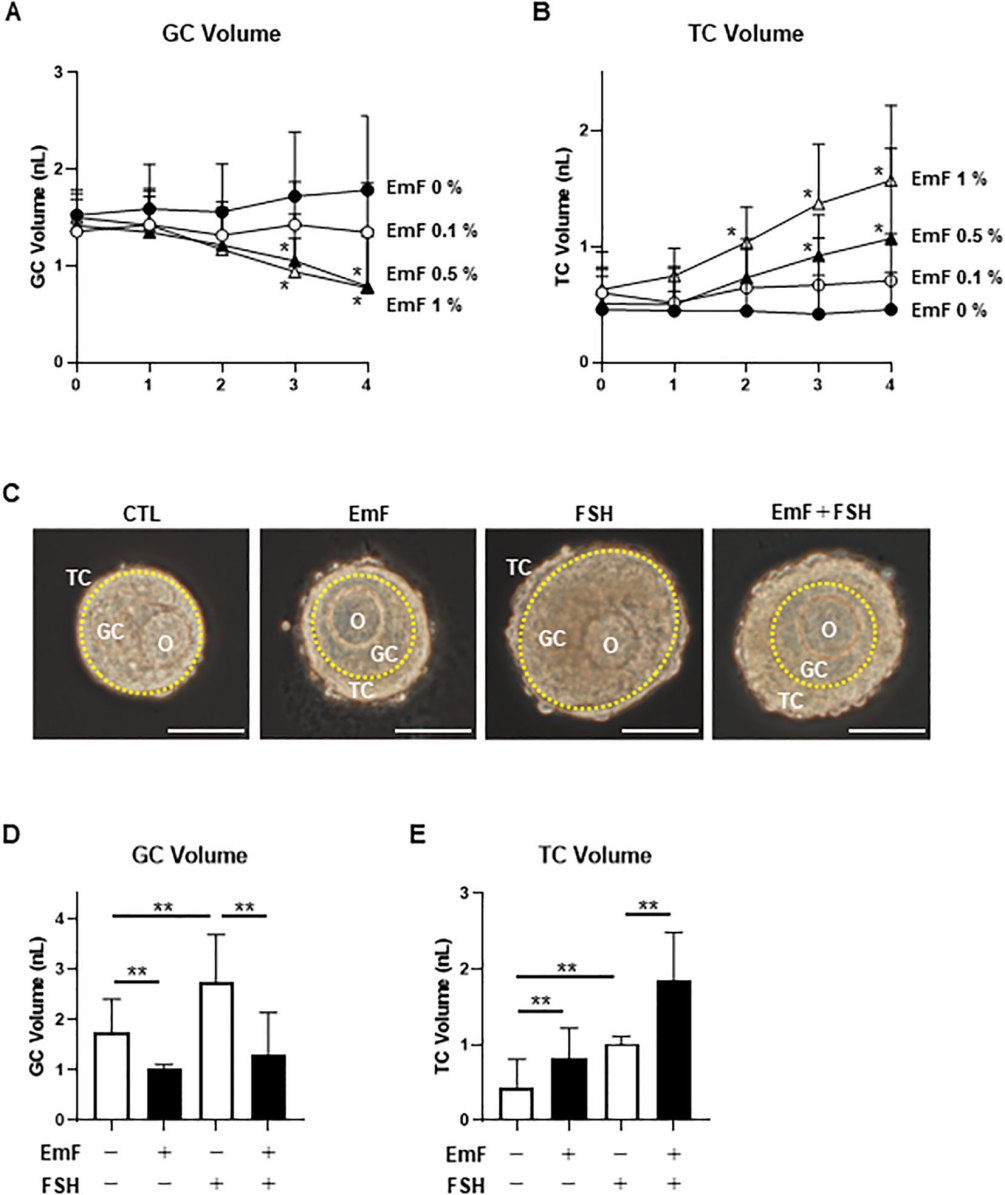
Effect of endometrioma fluid on the *in vitro* growth of preantral follicles. Rat large preantral follicles were cultured in media containing 0%, 0.1%, 0.5%, or 1% endometrioma fluid (EmF). **(A)** Changes in GC, granulosa cell volume over 4 days of culture. **(B)** Changes in TC, theca cell volume over 4 days of culture. Notably, exposure to 0.5% and 1% EmF suppressed GC proliferation while promoting TC proliferation from day 3 onward during preantral follicle development. Preantral follicles were cultured for 3 days in the presence or absence of 0.5% EmF and 10 ng/mL FSH. **(C)** Representative images of follicles on day 3 under each treatment condition: control (no EmF, no FSH; CTL), EmF, FSH, and EmF + FSH. Yellow dotted lines indicate the follicular basement membrane. Scale bar, 100 μm. **(D)** GC volume after 3 days of culture. **(E)** TC volume after 3 days of culture. EmF suppressed GC proliferation while promoting TC proliferation during preantral follicle development, regardless of the presence of FSH. Asterisks indicate statistically significant differences (*P < 0.05; **P < 0.01). O, oocyte; GC, granulosa cells; TC, theca cells; EmF, endometrioma fluid.

To evaluate the impact of endometrioma fluid (EmF) on preantral follicle development, rat large preantral follicles with an intact TC layer were cultured for 4 days in media containing 0%, 0.1%, 0.5%, or 1% EmF. No significant alterations in GC volume were observed under the 0.1% EmF condition. In contrast, follicles exposed to 0.5% and 1% EmF exhibited significant reductions in GC volume from day 3 onward (**Figure 1A**). Similarly, no significant effects on TC volume were detected at 0.1% EmF. However, a significant increase in TC volume was observed from day 2 under the 1% EmF condition and from day 3 under the 0.5% EmF condition (**Figure 1B**). Based on these findings, 0.5% EmF was determined to be the minimal effective concentration capable of inducing reproducible and measurable alterations in both GC and TC compartments in our culture system. Accordingly, we selected 0.5% EmF and a 3-day exposure period for all subsequent experiments.

**Figure 1C** presents representative images of follicles from each treatment group on day 3 of culture, in the presence or absence of 0.5% EmF and 10 ng/mL FSH. Under control conditions (no EmF, no FSH), GC and TC volumes were 1.74 ± 0.65 nL and 0.42 ± 0.36 nL, respectively (**Figures 1D, E**). In the absence of FSH, EmF significantly reduced GC volume compared with controls (1.74 ± 0.65 nL vs. 1.04 ± 0.47 nL, P < 0.01; **Figure 1D**). While FSH stimulation increased GC volume (P < 0.01), EmF markedly suppressed this FSH-driven GC expansion (2.75 ± 0.94 nL vs. 1.29 ± 0.85 nL, P < 0.01; **Figure 1D**).

In contrast, EmF significantly increased TC volume in the absence of FSH (0.44 ± 0.36 nL vs. 0.83 ± 0.40 nL, P < 0.01; **Figure 1E**). FSH also stimulated TC proliferation (P < 0.01), and this effect was further enhanced by EmF (1.02 ± 0.56 nL vs. 1.85 ± 0.63 nL, P < 0.01; **Figure 1E**). Collectively, these findings indicate that EmF inhibits GC proliferation while promoting TC proliferation during preantral follicle development.

### 3.2 Effect of endometrioma fluid on steroidogenesis in preantral follicles

**Figure 2:**
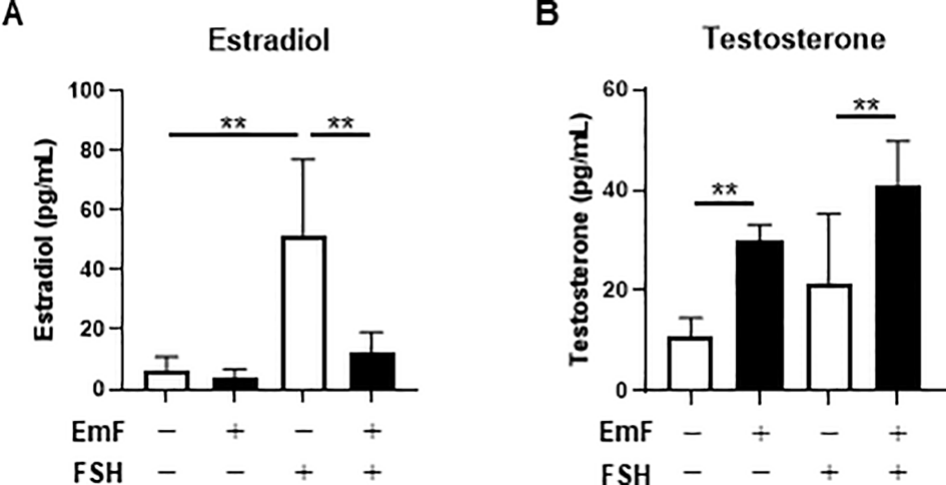
Effect of endometrioma fluid on steroidogenesis in preantral follicles. Concentrations of estradiol **(A)** and testosterone **(B)** in the spent media on day 3 were quantified. EmF markedly suppressed FSH-induced estradiol production, while enhancing testosterone production, regardless of FSH stimulation. Asterisks indicate statistically significant differences (**P < 0.01). EmF, endometrioma fluid.

To evaluate the influence of EmF on steroid hormone production, estradiol and testosterone concentrations in the culture media were measured. EmF had no effect on basal estradiol secretion but significantly suppressed FSH-induced estradiol production (**Figure 2A**). In contrast, EmF increased testosterone levels irrespective of FSH stimulation (**Figure 2B**).

**Figure 3:**
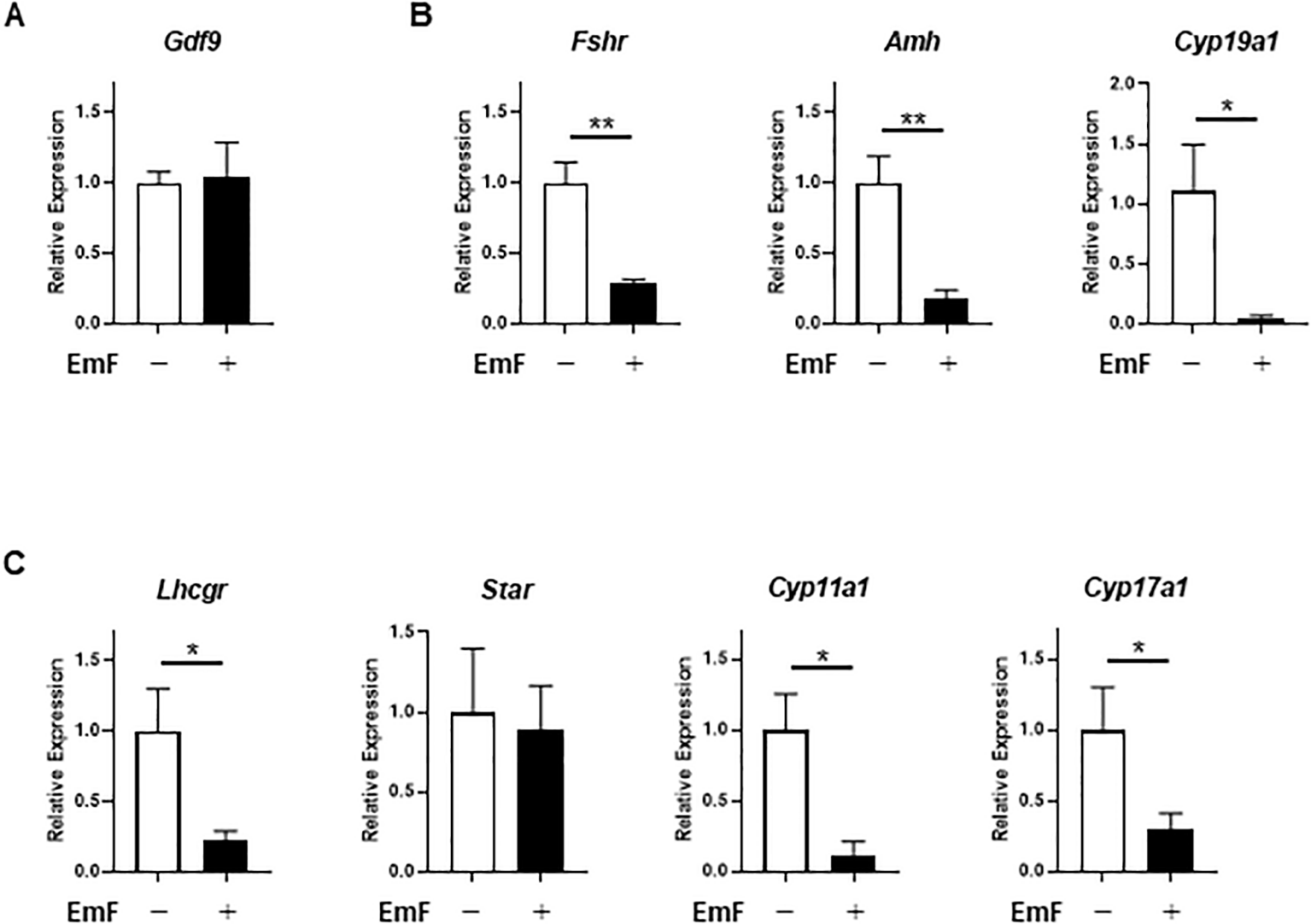
Effect of endometrioma fluid on gene expression in preantral follicles. **(A)** mRNA level of *Gdf9* (growth differentiation factor 9), an oocyte–related gene, was analyzed in the treated follicles on day 3. EmF did not affect *Gdf9* expression in preantral follicles. **(B)**, mRNA levels of granulosa cell-related genes, including *Fshr* (FSH receptor), *Amh* (anti-Müllerian hormone), and *Cyp19a1* (aromatase), were analyzed in the treated follicles on day 3. EmF significantly downregulated the expression of *Fshr*, *Amh*, and *Cyp19a1* in preantral follicles. **(C)**, mRNA levels of theca cell-related genes, including *Lhcgr* (LH receptor), *Star* (steroidogenic acute regulatory protein), *Cyp11a1* (cholesterol side-chain cleavage enzyme), and *Cyp17a1* (17α-hydroxylase/17,20-lyase), were analyzed in the treated follicles on day 3. EmF significantly suppressed the expression of *Lhcgr*, *Cyp11a1*, and *Cyp17a1* in preantral follicles. Asterisks indicate statistically significant differences (*P < 0.05; **P < 0.01). EmF, endometrioma fluid.

To examine transcriptional alterations induced by EmF, qRT-PCR was performed for genes predominantly expressed in the oocyte (*Gdf9*), GC (*Fshr*, *Amh*, *Cyp19a1*), and TC (*Lhcgr*, *Star*, *Cyp11a1*, and *Cyp17a1*). EmF exposure did not affect *Gdf9* expression in preantral follicles (**Figure 3A**). In contrast, EmF significantly downregulated *Fshr*, *Amh*, and *Cyp19a1* transcripts (**Figure 3B**). While *Star* expression remained unaffected, *Lhcgr*, *Cyp11a1*, and *Cyp17a1* mRNA levels were significantly reduced (**Figure 3C**). These results suggest that EmF impairs estrogenic activity in preantral follicles by suppressing GC function. Although EmF initially promoted TC proliferation and androgen production, it subsequently reduced the expression of key androgenic enzymes in the TC layer.

### 3.3 Effect of endometrioma fluid on oxidative stress in preantral follicles

**Figure 4:**
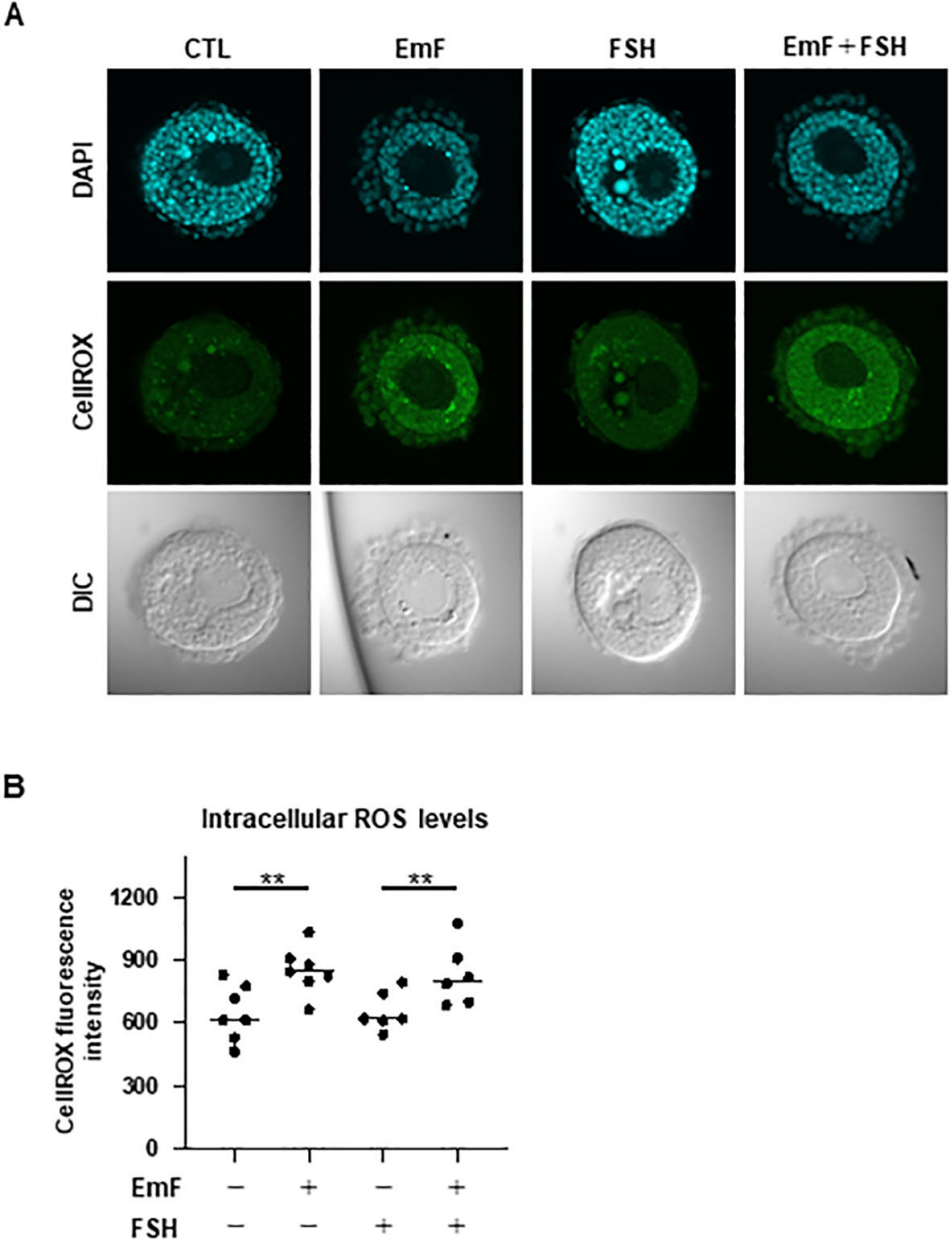
Effect of endometrioma fluid on oxidative stress in preantral follicles. Oxidative stress in preantral follicles was evaluated using CellROX Green Reagent to detect intracellular ROS, reactive oxygen species, with nuclei counterstained with DAPI. **(A)** Representative images on day 3 are shown for each treatment group: control (no EmF, no FSH; CTL), EmF, FSH, and EmF + FSH. **(B)** Intracellular ROS levels were quantified based on CellROX Green fluorescence intensity. EmF significantly elevated ROS accumulation within the granulosa cell layer of preantral follicles. Asterisks indicate statistically significant differences (**P < 0.01). EmF, endometrioma fluid; DIC, differential interference contrast; ROS, reactive oxygen species.

To determine whether EmF induces oxidative stress, intracellular ROS levels were assessed using CellROX Green Reagent. Confocal microscopy revealed a marked increase in ROS-related fluorescence within the GC layer of EmF-treated follicles, regardless of the presence of FSH (**Figures 4A, B**). These results indicate that EmF triggers oxidative stress in the GC layer of preantral follicles.

### 3.4 Effect of endometrioma fluid on tissue fibrosis in preantral follicles

**Figure 5:**
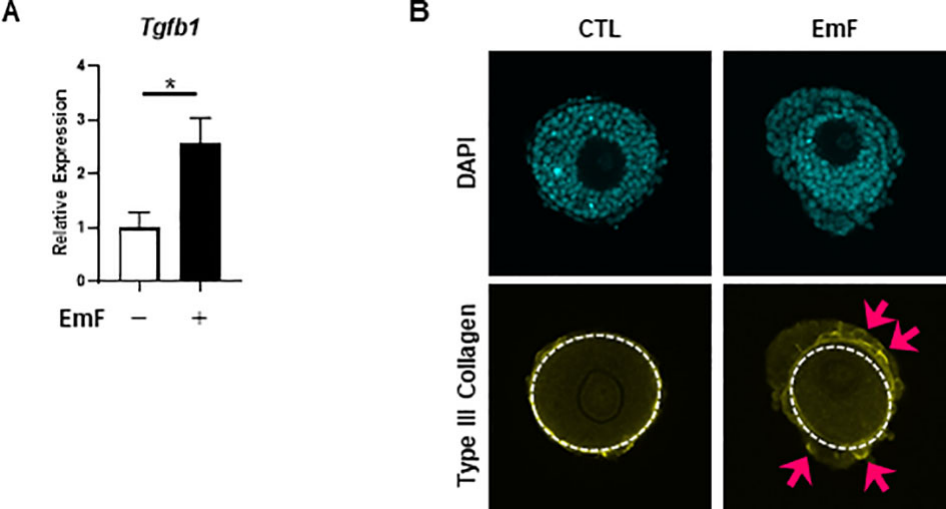
Effect of endometrioma fluid on tissue fibrosis in preantral follicles. **(A)** mRNA expression of *Tgfb1* (transforming growth factor beta 1), a pro-fibrotic marker, was analyzed in the treated follicles on day 3. EmF significantly upregulated *Tgfb1* expression in preantral follicles. **(B)** Follicular tissue fibrosis was evaluated by assessing type III collagen deposition. EmF markedly increased type III collagen accumulation within the theca cell layer. Asterisks indicate statistically significant differences (*P < 0.05). Pink arrows indicate type III collagen deposition. EmF, endometrioma fluid; CTL, control (no EmF, no FSH).

Given the observed loss of androgenic enzyme expression in the TC layer despite increased proliferation, we hypothesized that EmF might induce fibrotic remodeling. qRT-PCR analysis demonstrated a significant increase in the expression of *Tgfb1*, a pro-fibrotic marker, in EmF-treated follicles (**Figure 5A**). Immunofluorescence staining revealed increased type III collagen deposition, a marker of fibrosis, in the TC layer (**Figure 5B**). These findings suggest that EmF promotes tissue fibrosis in the TC layer of preantral follicles.

### 3.5 Pharmacological modulation of EmF-induced effects in preantral follicles

**Figure 6:**
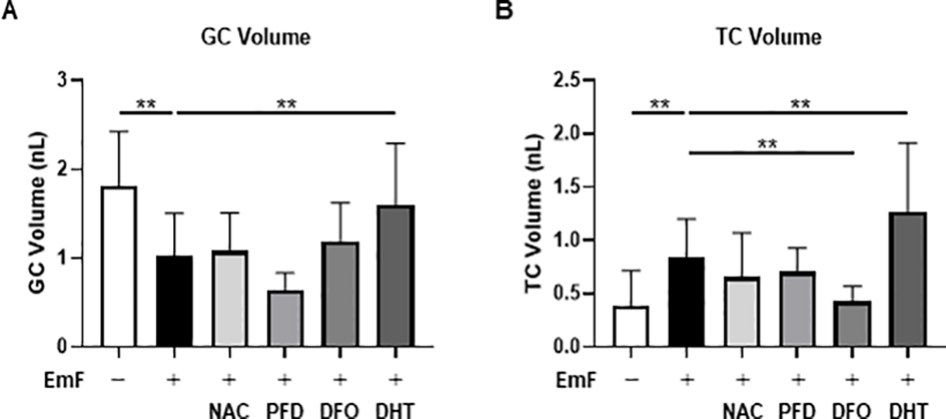
Pharmacological modulation of EmF-induced alterations in preantral follicle growth. To investigate potential therapeutic interventions, preantral follicles were co-treated with EmF and various agents, including N-acetyl-L-cysteine (NAC; antioxidant), pirfenidone (PFD; antifibrotic agent), deferoxamine mesylate (DFO; iron chelator), and dihydrotestosterone (DHT; non-aromatizable androgen). **(A)** GC, Granulosa cell volume after 3 days of culture. DHT effectively restored GC proliferation in EmF-treated follicles. **(B)** TC, Theca cell volume after 3 days of culture. EmF-induced TC expansion was suppressed by DFO but further enhanced by DHT. Asterisks indicate statistically significant differences (**P < 0.01). EmF, endometrioma fluid; GC, granulosa cells; TC, theca cells; NAC, N-acetyl-L-cysteine; PFD, pirfenidone; DFO, deferoxamine mesylate; DHT, dihydrotestosterone.

To explore potential therapeutic strategies, preantral follicles were co-treated with EmF and various pharmacological agents. EmF significantly reduced GC volume; however, neither NAC (an antioxidant), PFD (an antifibrotic agent), nor DFO (an iron chelator) reversed this inhibitory effect (**Figure 6A**). To further investigate the underlying mechanism, DHT (a non-aromatizable androgen) was introduced as a mechanistic probe to examine the role of TC–derived androgen signaling in regulating GC function. Under these conditions, DHT restored GC proliferation in EmF-treated follicles (1.06 ± 0.47 nL vs. 1.60 ± 0.69 nL, P < 0.01; **Figure 6A**). EmF-induced TC expansion was unaffected by NAC or PFD but was suppressed by DFO (0.85 ± 0.40 nL vs. 0.42 ± 0.14 nL, P < 0.01) and further enhanced by DHT (0.85 ± 0.40 nL vs. 1.27 ± 0.65 nL, P < 0.01; **Figure 6B**).

**Figure 7:**
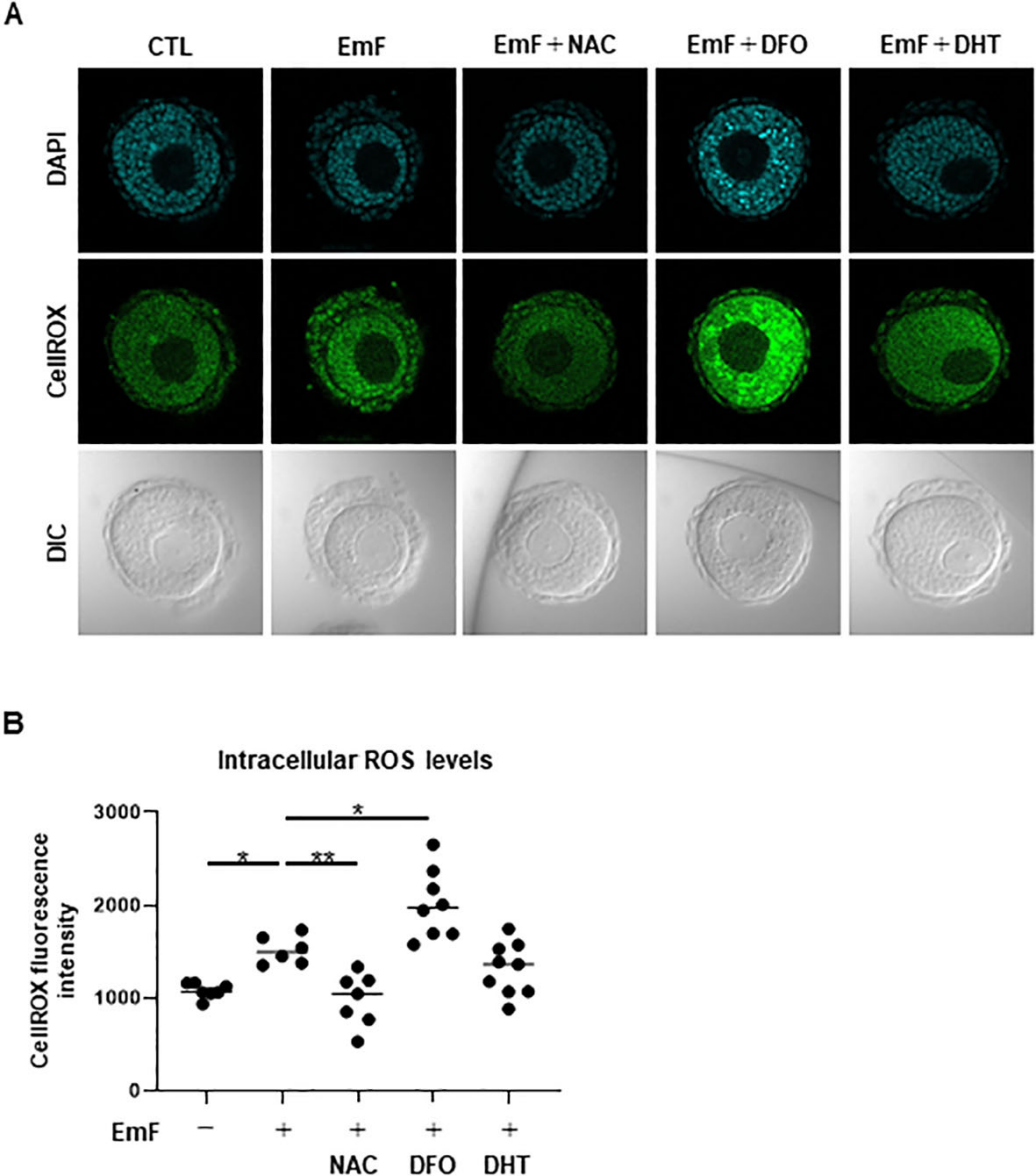
Pharmacological modulation of EmF-induced oxidative stress in preantral follicles. Oxidative stress was evaluated in preantral follicles using CellROX Green Reagent to detect intracellular ROS, reactive oxygen species, with nuclei counterstained with DAPI. **(A)**, Representative images on day 3 are shown for each treatment group: control (no EmF, no FSH; CTL), EmF, EmF + N-acetyl-L-cysteine (NAC; antioxidant), EmF + deferoxamine mesylate (DFO; iron chelator), and EmF + dihydrotestosterone (DHT; non-aromatizable androgen). **(B)** Intracellular ROS levels were quantified based on CellROX Green fluorescence intensity. EmF-induced ROS accumulation was effectively mitigated by NAC, remained unchanged with DHT, and was paradoxically exacerbated by DFO. Asterisks indicate statistically significant differences (*P < 0.05; **P < 0.01). EmF, endometrioma fluid; DIC, differential interference contrast; ROS, reactive oxygen species; NAC, N-acetyl-L-cysteine; DFO, deferoxamine mesylate; DHT, dihydrotestosterone.

**Figure 8:**
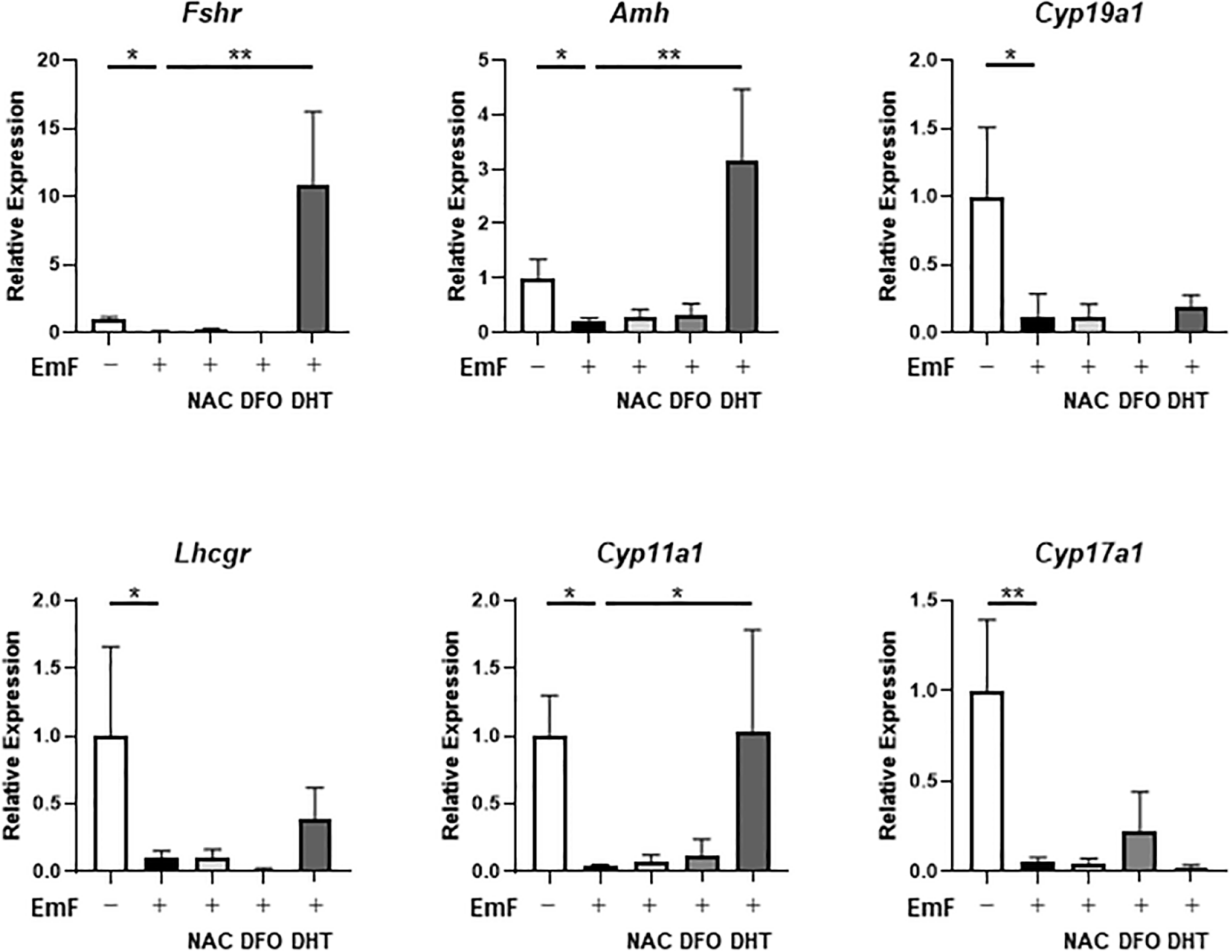
Pharmacological modulation of EmF-induced alterations in gene expression in preantral follicles. mRNA levels of granulosa cell–associated genes—*Fshr* (FSH receptor), *Amh* (anti-Müllerian hormone), and *Cyp19a1* (aromatase)—as well as theca cell–associated genes—*Lhcgr* (LH receptor), *Cyp11a1* (cholesterol side-chain cleavage enzyme), and *Cyp17a1* (17α-hydroxylase/17,20-lyase)—were analyzed in EmF-treated follicles on day 3. DHT treatment upregulated the mRNA levels of *Fshr*, *Amh*, and *Cyp11a1* in EmF-exposed follicles. Asterisks indicate statistically significant differences (*P < 0.05; **P < 0.01). EmF, endometrioma fluid; NAC, N-acetyl-L-cysteine (antioxidant); DFO, deferoxamine mesylate (iron chelator); DHT, dihydrotestosterone (non-aromatizable androgen).

EmF-mediated ROS accumulation in the GC layer was attenuated by NAC, unaffected by DHT, and exacerbated by DFO (**Figures 7A, B**). Moreover, DHT increased the mRNA levels of *Fshr*, *Amh*, and *Cyp11a1* in EmF-treated follicles, suggesting that androgens mitigate the EmF-induced reduction in FSH sensitivity (**Figure 8**).

## 4 Discussion

In this study, we investigated the effects of endometrioma fluid (EmF) on preantral follicle development using a rat follicle culture model. We found that EmF significantly reduced GC volume and *Fshr* expression and induced oxidative stress in the GC layer of preantral follicles. In contrast, EmF increased TC volume while concurrently downregulating the expression of key TC functional markers, suggesting that EmF promotes the proliferation of functionally compromised TC. Moreover, EmF upregulated *Tgfb1* and type III collagen expression in the TC layer, indicating the induction of fibrotic remodeling. Importantly, treatment with DHT, a non-aromatizable androgen, partially alleviated EmF-induced suppression of GC proliferation, suggesting that androgen signaling may counteract certain detrimental effects of EmF. Collectively, these findings demonstrate that EmF induces oxidative stress in GC and fibrosis in TC, thereby impairing preantral follicle development.

Endometriomas are known to compromise ovarian function through multiple, interrelated mechanisms. Structurally, the presence of an endometrioma induces architectural distortion and ovarian fibrosis, thereby physically restricting follicular development. Histological studies have reported reduced follicular density in the ovarian cortex adjacent to endometriomas (17). Biochemically, EmF contains elevated levels of pro-inflammatory cytokines, including IL-1β, IL-6, and TNF-α, as well as growth factors such as insulin-like growth factors (11). These bioactive components can activate signaling cascades, such as the PI3K/Akt pathway, in GC and TC, thereby accelerating premature follicular activation and atresia (18, 19).

In this study, we demonstrate that EmF induces oxidative stress in GC, thereby impairing GC function and suppressing preantral follicle development. Pro-inflammatory cytokines and iron-derived free radicals—both abundantly present in endometriomas—are potent inducers of ROS, leading to lipid peroxidation, protein oxidation, and DNA damage (10, 20). During the preantral-to-early antral transition, acquisition of FSH responsiveness is essential for sustained follicular growth and survival. In the present study, EmF markedly suppressed *Fshr* expression in preantral follicles and attenuated FSH-induced GC proliferation and estradiol production. Collectively, these findings indicate that EmF disrupts the establishment of FSH dependency, thereby compromising the developmental competence of preantral follicles. Taken together, our results identify EmF-induced oxidative stress in GC as a previously unrecognized mechanism by which ovarian endometriomas impair ovarian function in endometriosis-associated infertility.

A key unresolved question is whether EmF directly induces oxidative stress in GC or indirectly impairs GC function by disrupting paracrine signaling from TC. The bidirectional interaction between GC and TC is essential for follicular growth, gonadotropin responsiveness, and survival during the preantral-to-antral transition (21, 22). TC-derived androgens and growth factors play crucial roles in supporting GC proliferation, steroidogenesis, and anti-apoptotic pathways (23–25). Although EmF stimulated TC proliferation, these hypertrophied TC exhibited reduced expression of key androgenic enzymes. Notably, DHT partially restored GC proliferation and FSH responsiveness in EmF-treated follicles, suggesting that EmF disrupts TC-mediated paracrine support. In parallel, recent studies have demonstrated that excessive ROS in endometriosis can directly induce ER stress-mediated senescence in GC, thereby contributing to endometriosis-associated infertility (26). Taken together, these findings support the possibility that EmF impairs GC function through both direct induction of oxidative stress and indirect disruption of TC-derived paracrine signaling.

Pro-inflammatory cytokines and free iron are also well-known drivers of tissue fibrosis (11, 27). Excessive ROS can amplify inflammatory signaling, thereby perpetuating tissue injury and fibrotic remodeling within the ovarian microenvironment. Follicular development is highly sensitive to alterations in the mechanical stiffness of the surrounding tissue (28). Histologically, endometriomas are characterized by extensive fibrosis within and surrounding the cyst wall (29). In the present study, EmF induced collagen deposition in the TC layer, potentially altering its biomechanical properties. Increased tissue stiffness resulting from fibrosis can impair follicular expansion and development through mechanotransduction pathways (30, 31). Accordingly, EmF-induced TC fibrosis may represent an additional mechanism by which preantral follicle development is compromised.

Our findings suggest that targeting EmF-induced oxidative stress in GC and fibrotic changes in TC may represent a potential strategy for restoring ovarian function in endometriosis-associated infertility. Previous animal studies have reported that antioxidants (32, 33), antifibrotic agents (34), and iron chelators (35) can attenuate follicular damage. In our experiments, however, neither antifibrotic agents nor iron chelators were able to reverse the deleterious effects of EmF on preantral follicle development. Although antioxidant treatment effectively reduced intracellular ROS levels, it failed to aid in follicular growth.

In contrast, DHT partially counteracted the EmF-induced suppression of GC proliferation and FSH responsiveness. Notably, DHT was employed exclusively as a mechanistic probe to elucidate the role of TC-derived androgen signaling in regulating GC function, rather than as a therapeutic agent, as exogenous androgen administration is not a clinically appropriate option for infertile women. These findings highlight the multifactorial nature of EmF-induced follicular damage and suggest that effective interventions may require combined strategies targeting oxidative stress, fibrosis, and disrupted paracrine signaling.

Our findings should be interpreted in the context of existing clinical evidence. Women with ovarian endometriomas undergoing ART generally achieve comparable live birth and clinical pregnancy rates to those without endometriomas, despite reduced oocyte yield (36). Surgical treatment does not consistently improve ART outcomes and may further compromise ovarian reserve, with its impact varying depending on surgical and hemostatic techniques (37–39). These observations highlight a complex clinical dilemma, as both the persistence of endometriomas and their surgical removal carry potential risks to ovarian function. While our study does not advocate a specific management strategy, it provides mechanistic insight into how endometrioma-derived factors may disrupt the ovarian microenvironment and contribute to reduced oocyte yield despite preserved ART outcomes.

Currently, there is no definitive clinical evidence that removal of EmF improves ovarian function or reproductive outcomes. However, growing interest in minimally invasive approaches, such as ethanol sclerotherapy, suggests the potential to improve the local ovarian environment while preserving ovarian reserve (40–43). Our findings support this concept by indicating that removal or neutralization of EmF may reduce inflammatory and oxidative stress within the ovary. These results further suggest that ovary-preserving strategies—such as EmF drainage combined with localized pharmacological interventions—may mitigate oxidative stress and fibrosis without inducing surgical damage. Whether such approaches can improve reproductive outcomes remains to be established and warrants further investigation.

This study has certain limitations. First, EmF samples were pooled from six patients, which may not fully capture inter-patient heterogeneity. Given the known variability in disease severity, inflammatory profiles, and ovarian reserve among women with ovarian endometriomas, the biochemical composition and biological activity of EmF are likely to differ between individuals. Notably, not all women with ovarian endometriomas exhibit impaired ovarian function, suggesting that the detrimental effects of EmF may vary across endometrioma subtypes or patient-specific conditions. Therefore, evaluating the effects of EmF derived from individual patients represents an important priority for future studies to validate the consistency and clinical relevance of the observed findings. Second, although EmF was tested at a concentration of 0.5% *in vitro*, the actual *in vivo* concentration of toxic components and their diffusion into the adjacent ovarian cortex remain unknown. At present, there are no available data—either from previous studies or from our own—to estimate the extent to which EmF-derived factors permeate follicles located near ovarian endometriomas *in vivo*. Although prolonged exposure to lower EmF concentrations might more closely reflect physiological conditions, the constraints of our serum-free *in vitro* follicle culture system necessitated the use of relatively higher EmF concentrations over a shorter exposure period to elicit reproducible and quantifiable biological responses.

Another important limitation concerns the identity of the specific bioactive molecule(s) within EmF responsible for the observed follicular alterations. Nevertheless, previous studies have consistently reported markedly elevated levels of pro-inflammatory cytokines and free iron in follicular fluid adjacent to endometriomas (2, 11, 44, 45). In this context, we have recently observed that (i) TNF-α induces oxidative stress in GC and suppresses GC proliferation, and (ii) IL-1β and IL-6 promote non-functional proliferation and fibrotic remodeling in TC (46). Although EmF contains a variety of bioactive components beyond inflammatory cytokines, these overlapping phenotypes suggest that pro-inflammatory cytokines may play a central role in mediating the detrimental effects observed in this study. In addition, the potential impact of excessive iron contained in EmF on follicular development warrants further investigation.

Finally, immune or stromal components within the ovarian microenvironment may mitigate the harmful effects of EmF *in vivo*, a factor that cannot be fully recapitulated in our *in vitro* model. Further studies using human ovarian tissue and patient-stratified analyses are therefore needed to validate these findings and to identify subgroups of patients who may be particularly susceptible to EmF-induced follicular damage.

In summary, our study provides novel mechanistic evidence that endometrioma-derived factors impair early follicular development through the combined effects of oxidative stress and tissue remodeling. These findings highlight the critical role of the local ovarian microenvironment in endometriosis-associated infertility and support the need for individualized, mechanism-based therapeutic strategies.

## Data Availability

The raw data supporting the conclusions of this article will be made available by the authors, without undue reservation.
