# Supplementary material for: Negative impacts of ovarian endometrioma on preantral follicle development: implications for endometriosis-related infertility
Source: Front Endocrinol (Lausanne). 2026 May 11;17:1679042. doi: 10.3389/fendo.2026.1679042 (PMC13199029; doi:10.3389/fendo.2026.1679042)
Supplement: Supplementary file 1 [file Presentation1.pptx]

## Slide 1
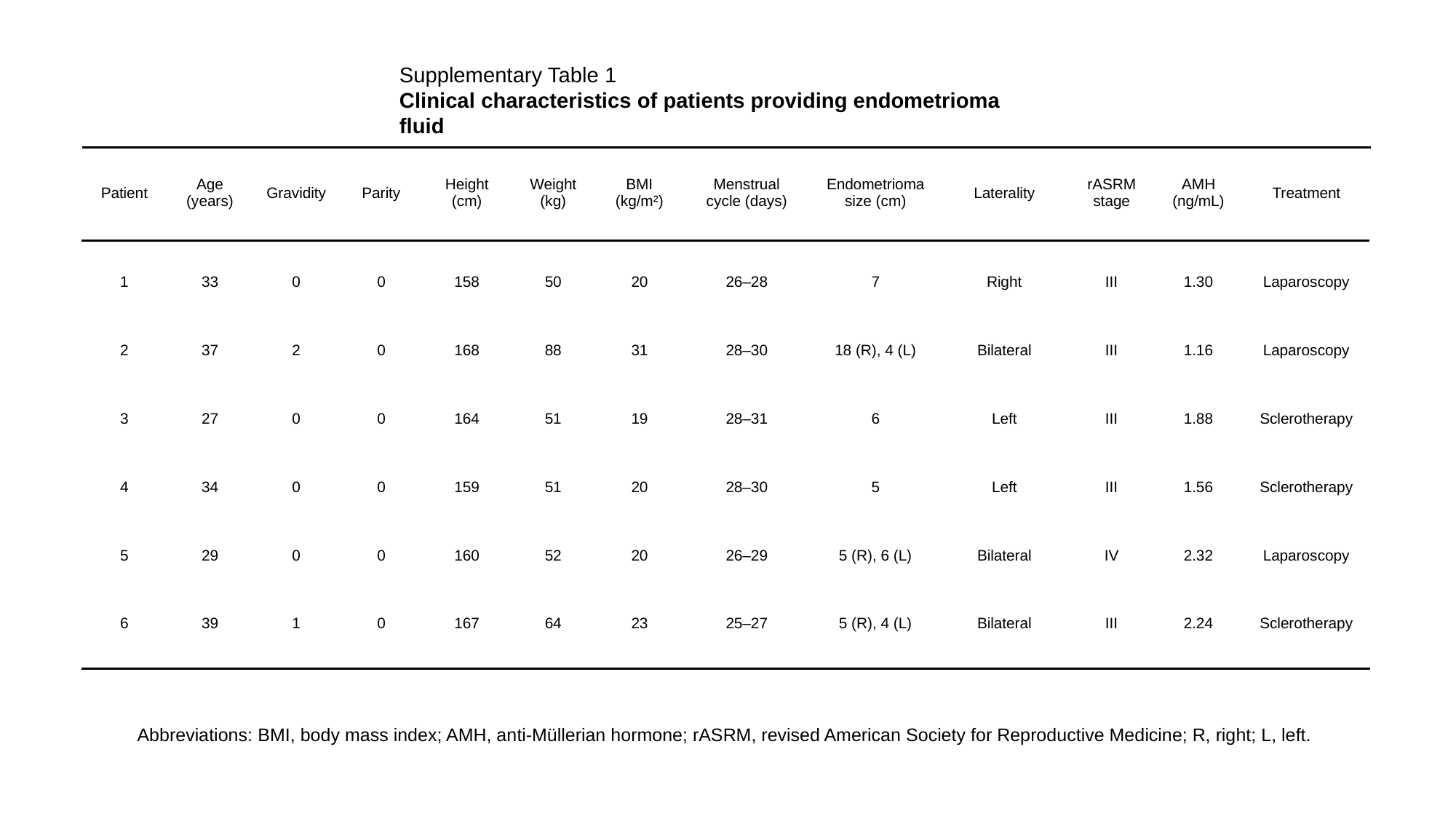

Supplementary Table 1
Clinical characteristics of patients providing endometrioma fluid
| Patient | Age (years) | Gravidity | Parity | Height (cm) | Weight (kg) | BMI (kg/m²) | Menstrual cycle (days) | Endometrioma size (cm) | Laterality | rASRM stage | AMH (ng/mL) | Treatment |
| --- | --- | --- | --- | --- | --- | --- | --- | --- | --- | --- | --- | --- |
| 1 | 33 | 0 | 0 | 158 | 50 | 20 | 26–28 | 7 | Right | III | 1.30 | Laparoscopy |
| 2 | 37 | 2 | 0 | 168 | 88 | 31 | 28–30 | 18 (R), 4 (L) | Bilateral | III | 1.16 | Laparoscopy |
| 3 | 27 | 0 | 0 | 164 | 51 | 19 | 28–31 | 6 | Left | III | 1.88 | Sclerotherapy |
| 4 | 34 | 0 | 0 | 159 | 51 | 20 | 28–30 | 5 | Left | III | 1.56 | Sclerotherapy |
| 5 | 29 | 0 | 0 | 160 | 52 | 20 | 26–29 | 5 (R), 6 (L) | Bilateral | IV | 2.32 | Laparoscopy |
| 6 | 39 | 1 | 0 | 167 | 64 | 23 | 25–27 | 5 (R), 4 (L) | Bilateral | III | 2.24 | Sclerotherapy |
Abbreviations: BMI, body mass index; AMH, anti-Müllerian hormone; rASRM, revised American Society for Reproductive Medicine; R, right; L, left.
