# Supplementary material for: Negative impacts of ovarian endometrioma on preantral follicle development: implications for endometriosis-related infertility
Source: Front Endocrinol (Lausanne). 2026 May 11;17:1679042. doi: 10.3389/fendo.2026.1679042 (PMC13199029; doi:10.3389/fendo.2026.1679042)
Supplement: Supplementary file 2 [file Presentation2.pptx]

## Slide 1
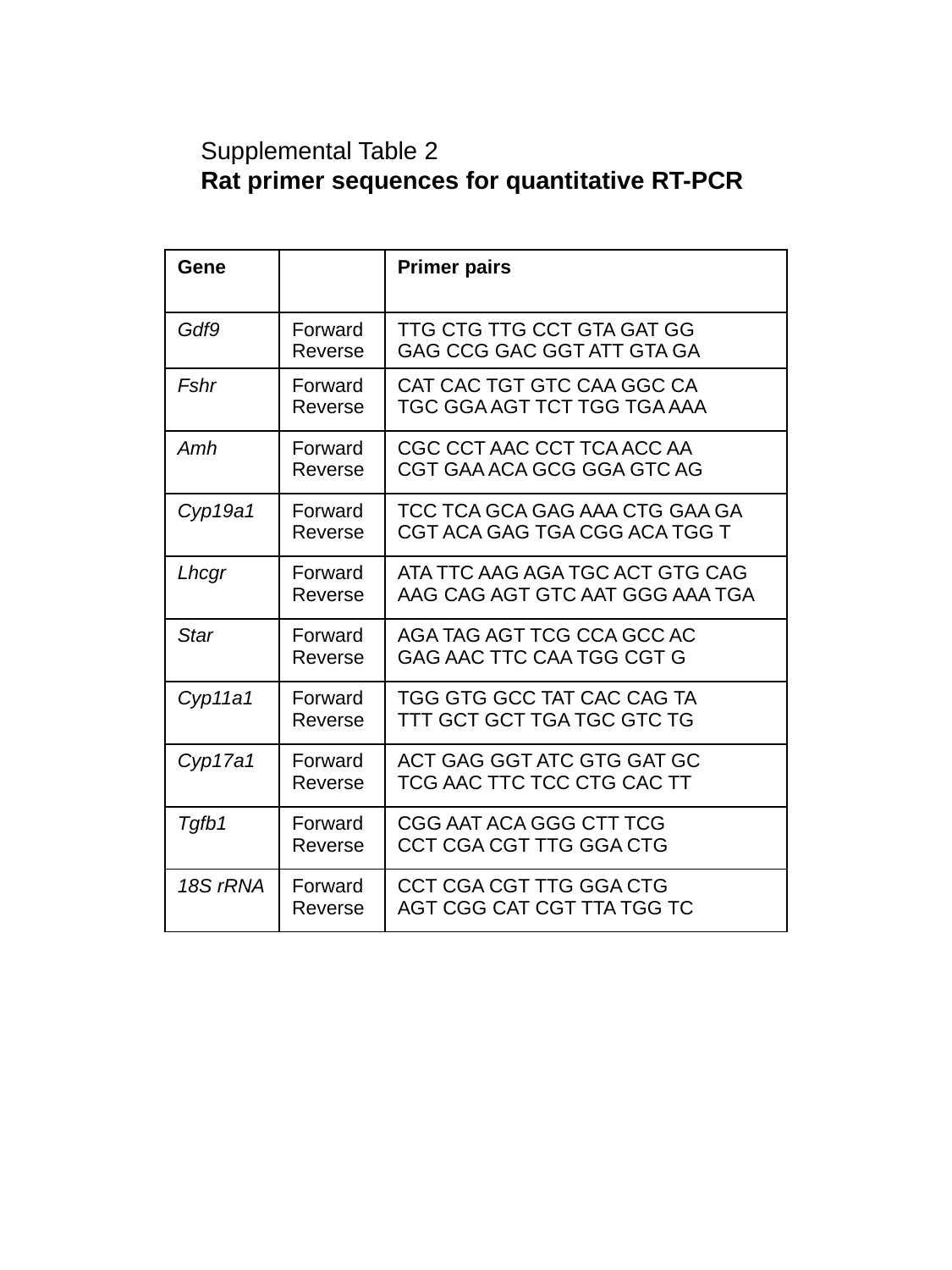

Supplemental Table 2
Rat primer sequences for quantitative RT-PCR
| Gene | | Primer pairs |
| --- | --- | --- |
| Gdf9 | Forward Reverse | TTG CTG TTG CCT GTA GAT GG GAG CCG GAC GGT ATT GTA GA |
| Fshr | Forward Reverse | CAT CAC TGT GTC CAA GGC CA TGC GGA AGT TCT TGG TGA AAA |
| Amh | Forward Reverse | CGC CCT AAC CCT TCA ACC AA CGT GAA ACA GCG GGA GTC AG |
| Cyp19a1 | Forward Reverse | TCC TCA GCA GAG AAA CTG GAA GA CGT ACA GAG TGA CGG ACA TGG T |
| Lhcgr | Forward Reverse | ATA TTC AAG AGA TGC ACT GTG CAG AAG CAG AGT GTC AAT GGG AAA TGA |
| Star | Forward Reverse | AGA TAG AGT TCG CCA GCC AC GAG AAC TTC CAA TGG CGT G |
| Cyp11a1 | Forward Reverse | TGG GTG GCC TAT CAC CAG TA TTT GCT GCT TGA TGC GTC TG |
| Cyp17a1 | Forward Reverse | ACT GAG GGT ATC GTG GAT GC TCG AAC TTC TCC CTG CAC TT |
| Tgfb1 | Forward Reverse | CGG AAT ACA GGG CTT TCG CCT CGA CGT TTG GGA CTG |
| 18S rRNA | Forward Reverse | CCT CGA CGT TTG GGA CTG AGT CGG CAT CGT TTA TGG TC |
